# Wernicke Encephalopathy in a Child With Acute Lymphoblastic Leukemia: A Case Report

**DOI:** 10.1002/cnr2.70235

**Published:** 2025-06-23

**Authors:** Ghazaleh Shakibamaram, Mohammadreza Dolikhani, Farideh Moussavi, Syna Sarraf, Bredsin Benyamin, Soroor Advani

**Affiliations:** ^1^ Medical School of Shahid Beheshti University of Medical Sciences Tehran Iran; ^2^ Pediatric Department Shohada‐e Tajrish Hospital, Shahid Beheshti University of Medical Sciences Tehran Iran; ^3^ Clinical Research Development Unit Shohada‐e Tajrish Hospital, Shahid Beheshti University of Medical Sciences Tehran Iran; ^4^ Neurology Department Shohada‐e Tajrish Hospital, Shahid Beheshti University of Medical Sciences Tehran Iran

**Keywords:** acute lymphoblastic leukemia, pediatric oncology, thiamine deficiency, Wernicke encephalopathy

## Abstract

**Background:**

Wernicke encephalopathy (WE) is a life‐threatening neurological disorder caused by thiamine deficiency, commonly associated with alcoholism but also observed in malnourished pediatric cancer patients undergoing intensive chemotherapy. WE remains underdiagnosed in children, with many cases only confirmed postmortem. We report a 6‐year‐old girl with acute lymphoblastic leukemia (ALL) who developed WE secondary to treatment‐resistant nausea and vomiting.

**Case:**

The patient presented with acute gait disturbance, ophthalmoparesis, and paraparesis following persistent vomiting and significant weight loss. Initial diagnostic evaluations, including cerebrospinal fluid analysis and neuroimaging, suggested alternative diagnoses such as cerebellitis and Guillain‐Barré Syndrome. However, progressive neurological deterioration, the emergence of encephalopathy, and follow‐up magnetic resonance imaging (MRI) findings of hyperintense lesions in the periventricular, periaqueductal, and cerebellar regions supported the diagnosis of WE. The overlapping features with other neurological conditions contributed to a delay in recognizing WE and initiating thiamine therapy. Despite initiating high‐dose intravenous thiamine, symptom resolution was significant but partial. Unfortunately, the patient later developed lymphomatous meningitis and sepsis and ultimately succumbed to complications.

**Conclusion:**

This case highlights the importance of early clinical recognition of WE in pediatric leukemia patients with prolonged vomiting, as delayed diagnosis can lead to irreversible neurological damage or death. Given the limitations of early neuroimaging findings, clinical suspicion should prompt immediate thiamine supplementation. The report points out the need for heightened awareness of thiamine deficiency in pediatric oncology, emphasizing the role of prophylactic supplementation in high‐risk patients.

**Abbreviations**
ALL: acute lymphoblastic leukemia
MRI: magnetic resonance imaging
WE: Wernicke encephalopathy

## 1 Introduction

Wernicke encephalopathy (WE) is a life‐threatening disorder caused by thiamine (vitamin B1) deficiency [1]. Alcoholism remains the leading cause of WE [1, 2]. Other etiologies (such as malnutrition, bariatric surgery, prolonged parenteral nutrition, starvation, and severe and prolonged vomiting) are less frequent but clinically important [1, 3].

WE is often underdiagnosed in children, with postmortem examinations confirming the diagnosis in about one‐third of pediatric cases [4]. Thiamine deficiency in pediatric cancer patients, especially those undergoing intensive treatments for leukemia or solid tumors, is caused by malnutrition, vomiting, malabsorption, the effects of chemotherapy, and so on [5]. Certain chemotherapy drugs, such as cytarabine, can inactivate thiamine [5, 6]. There are only a few reports in the literature addressing WE in pediatric cancer patients [7, 8, 9, 10].

The clinical trial of WE is confusion, ataxia, and ophthalmoplegia, seen only in 10% of patients [11, 12]. The most common symptom is changing mental status (34%–82% of patients) with the involvement of the mammillary bodies or reticular system [12]. Gait ataxia originates from vestibular or vermis dysfunction [12, 13]. WE diagnosis is clinically based and should be considered in patients with the abovementioned conditions [14]. Because WE is a medical emergency, high‐dose intravenous thiamine should be started immediately in patients with clinical suspicion to improve prognosis and decrease comorbidities [15].

Regarding neuroimaging, computed tomography (CT) scans mostly show nothing in the acute phase. Magnetic resonance imaging (MRI) is usually mandatory to confirm the diagnosis [2, 16]. If WE is not treated correctly, Korsakoff's psychosis or death may occur. Korsakoff syndrome presents with anterograde amnesia (lesions in mammillary bodies, anterior thalamic circuitry, or/mammillo‐thalamic tract), recall and recognition memory disorders, and short‐term forgetfulness [17]. The superior cerebellar vermis has seen a significant decline in Purkinje cells and a loss of neurons from the granular layer [18].

This report presents a 6‐year‐old child with acute lymphoblastic leukemia (ALL) who developed Korsakoff syndrome secondary to treatment‐resistant nausea and vomiting. In contrast, a limited number of WE cases in ALL have been documented internationally [19, 20, 21]. To the best of our knowledge, this is the first such case reported from Iran. This highlights the importance of raising regional awareness regarding this rare but serious complication in pediatric oncology.

## 2 Patient Presentation

A 6‐year‐old girl, born to consanguineous parents with a three‐year history of pure B‐cell ALL, developed severe nausea and vomiting 15 days after her most recent chemotherapy session for central nervous system (CNS) relapse. The patient was hospitalized for chemotherapy at Shohada‐e Tajrish Hospital of Tehran in December 2022. She had anorexia and 6 kg weight loss (38–32 kg in 2 months). At the time of admission for her chemotherapy, she still had nausea and vomiting and refused to eat any food. After 2 days, she developed acute gait disturbance due to an imbalance, followed by paraparesis after 2 days. There was no history of fever, loss of consciousness, speech, or visual impairment such as blurred or double vision, sensory, respiratory, swallowing, or sphincter impairment.

At the age of 3, the patient was diagnosed with ALL and underwent an 18‐month chemotherapy regimen, including methotrexate (MTX) (2000 mg/m^2^/day), asparaginase (10 000 U/m^2^/day), vincristine (1.5 mg/m^2^/day), cyclophosphamide (1000 mg/m^2^/day), and cytarabine (3 mg/m^2^/day), without significant complications and was administered according to the Berlin‐Frankfurt‐Münster (BFM) protocol.

During the maintenance phase of therapy, 8 months after initiation, she presented with complications of excessive appetite and an 18‐kg weight gain (from 20 to 38 kg). She was evaluated for CNS relapse due to suspected hypothalamic involvement. Brain MRI revealed bilateral signal changes in the anterior horns of the lateral ventricles, internal capsule, and left globus pallidus, while the hypothalamus appeared normal. Cerebrospinal fluid (CSF) analysis showed pleocytosis (white blood cell [WBC]: 50 cells/μL, lymphocytes 70%, PMN 30% [normal: 0–5 cells/μL/all mononuclear]), hypoglycorrhachia (glucose: 16 mg/dL [normal: 50–80 mg/dL]), and elevated RBCs (*N* = 2250 cells/μL [normal = 0 cells/μL]), leading to a diagnosis of CNS relapse. She was treated with 5 sessions of chemotherapy and 10 sessions of radiotherapy. Recently, hepatic evaluation for jaundice and elevated aspartate aminotransferase (AST) = 135 IU/I (normal = 10–40 IU/I) and alanine aminotransferase (ALT) = 159 IU/I (normal = 10–40 IU/I) ruled out viral hepatitis, with liver function normalizing before her subsequent chemotherapy session 2 weeks later.

Apart from the chemotherapy drugs, the patient used Cotrimoxazole syrup, 5 mL per day. Her allergy, family, surgical, and social histories were negative.

On examination, the patient's vital signs were stable and within normal ranges. No signs of meningeal irritation were observed, and Redor, Kernig, and Brudzinski tests were negative. Neurologically, she was awake, alert, oriented, and able to follow three‐step commands with normal speech. Bilateral esotropia was noted, with a 2+ limitation in outward gaze, but no nystagmus was present. Other cranial nerve functions were normal.

Motor examination revealed no atrophy or fasciculations. Muscle strength was 5/5 in the upper extremities and 2/5 in the lower extremities. Deep tendon reflexes (DTRs) were 2+ in the upper extremities but absent in both knees and Achilles tendons. Sensory examination was intact without any sensory level. Cerebellar testing showed dysmetria on the right side in finger‐to‐nose and heel‐to‐shin maneuvers. She was unable to walk independently but could sit without assistance.

There were no signs of jaundice or hepatomegaly despite her recent history of liver dysfunction. A maculopapular morbilliform rash, likely drug‐induced, appeared on the arms and back 26 days after admission, with no mucosal involvement.

### 2.1 Diagnostic Assessment

Based on her past medical history and clinical findings, a broad differential diagnosis was considered for acute flaccid paraparesis with absent lower limbs' DTRs and ophthalmoparesis. These included Miller–Fisher/Guillain–Barré Syndrome (GBS), infectious or neoplastic polyneuropathy, drug‐induced neurotoxicity, metabolic encephalopathies, paraneoplastic syndromes, brainstem stroke, and metastatic or infectious involvement of the brainstem or cerebellum. To exclude these possibilities, an extensive diagnostic workup was conducted.

**FIGURE 1:**
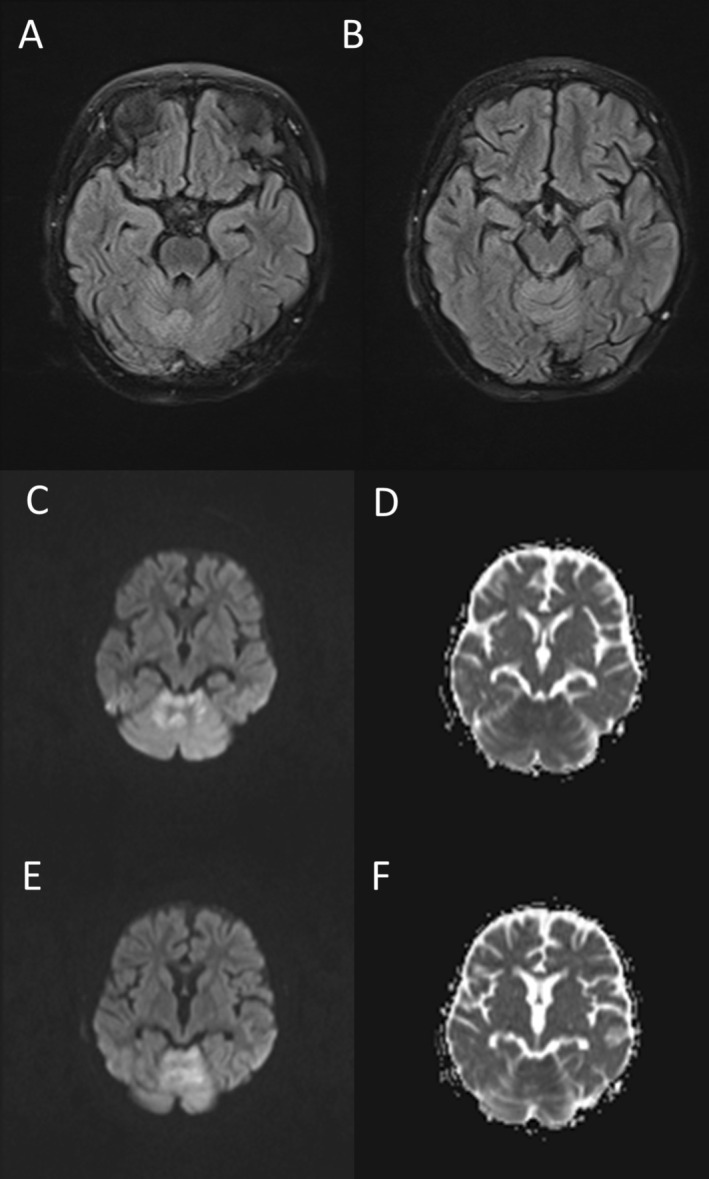
First MRI: Fluid attenuated inversion recovery (FLAIR). MRI showing hyperintensities in cerebellum (A, B). Restricted diffusion in cerebellum (C–F).

As per institutional protocol for new‐onset neurological symptoms during chemotherapy, the patient underwent an initial brain MRI with contrast before neurology consultation. This imaging revealed restricted diffusion in the cerebellar vermis, medial cerebellar hemispheres with a cortical pattern, and cerebellar peduncles. Based on these findings, a presumptive diagnosis of cerebellitis was made (Figure 1).

A lumbar puncture (LP) was subsequently performed to evaluate viral or bacterial involvement. CSF analysis revealed WBC < 5 cells/μL (normal = 0–5 cells/μL), RBC = 550 cells/μL (normal < 1 cell/μL), glucose 67 mg/dL (normal = 50–80 mg/dL), protein 23 mg/dL (normal = 15–40 mg/dL), and lactate dehydrogenase (LDH) 34 U/L (normal ≤ 40 U/L). Bacterial, viral, and fungal panels of CSF were negative.

**FIGURE 2:**
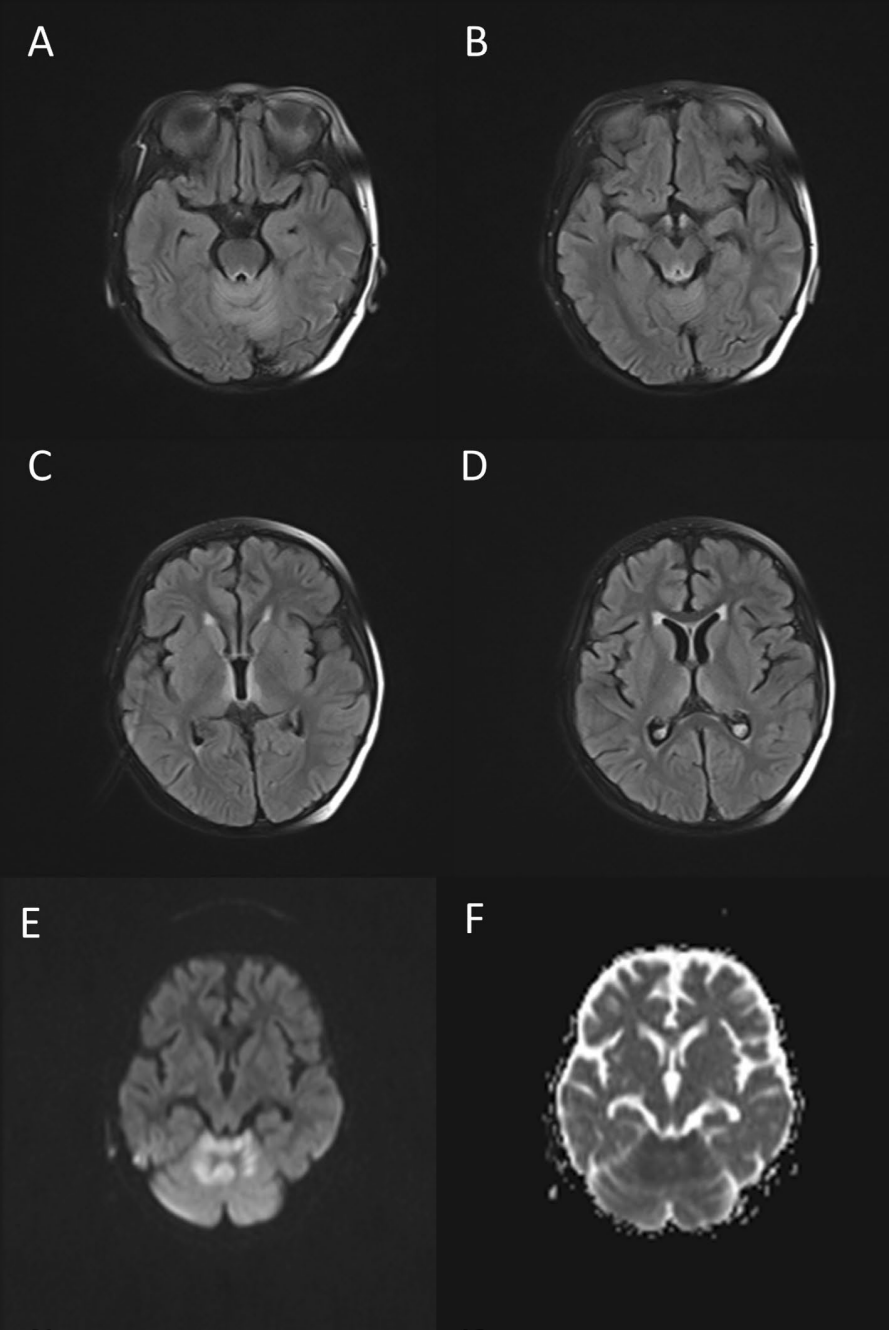
Second MRI: Fluid‐attenuated inversion recovery (FLAIR). MRI showing hyperintensities in the cerebellum, peri‐aqueductal region, and medial thalamic region (A–D). Restricted diffusion more evident in cerebellum (E, F).

Due to paraparesis, she was started on intravenous immunoglobulin (IVIg) for a suspected diagnosis of GBS. However, her symptoms progressed, and encephalopathy with confusion emerged. A second brain MRI was performed at this time, which revealed increased T2/FLAIR signal intensity around the lateral and third ventricles, periaqueductal gray matter, and superior cerebellar regions, with restricted diffusion but no meningeal enhancement (Figure 2). Electrodiagnostic studies demonstrated sensory and motor axonal polyneuropathy and no evidence of demyelinating features in favor of GBS. These findings led to the correct diagnosis of WE, and appropriate treatment was initiated, and IVIg was discontinued.

### 2.2 Therapeutic Intervention

During hospitalization, the patient was treated with intravenous thiamine (500 mg TDS for 15 days, then 100 mg daily), plus vitamins A, C, D_3_, folic acid, zinc, magnesium citrate, and an amino acid supplement. Her ophthalmoparesis was the first symptom to resolve. Cetirizine, betamethasone ointment, and Eucerin ointment were prescribed for her rash.

Bone marrow aspiration demonstrated complete remission in trilineage hematopoiesis. Persistent vomiting led to an endoscopy, which revealed mild reflux esophagitis, erosive gastritis, and prolapse gastropathy. Domperidone, pantoprazole, and sucralfate were given to address the gastrointestinal findings observed during the endoscopy. Echocardiography showed normal cardiac function with an ejection fraction of 55%–60%.

At discharge, she was prescribed oral vitamin B1 (300 mg daily), folic acid (10 mg daily), haloperidol (0.5 mg twice daily), cotrimoxazole syrup (5 mL every other day), zinc sulfate syrup (10 mL daily), and intramuscular B12 (1000 μg every week). By the time of discharge, she was able to walk with assistance.

### 2.3 Follow‐Up and Outcomes

About a month after discharge and completing three chemotherapy sessions, the patient presented to the emergency department with fever, tonic–clonic seizures, upward gaze, posturing (flexion of the upper limbs and extension of the lower limbs), and a post‐ictal state with stupor. Examination revealed a Glasgow Coma Scale (GCS) score of 8/15, limb strength of 2/5, and reduced tone and DTRs. She was intubated prophylactically due to a decreased level of consciousness and abundant nasopharyngeal secretions.

Brain MRI showed bilateral diffuse nodular leptomeningeal enhancement and cortical restriction in the frontotemporal regions. There was no evidence of the previous WE typical or atypical lesions. LP revealed CSF findings of WBC 70 cells/μL (normal = 0–5 cells/μL), glucose 10 mg/dL (normal = 50–80 mg/dL), protein 125 mg/dL (normal = 15–40 mg/dL), LDH 163 U/L (normal ≤ 40 U/L), and positive cultures for *Candida*, consistent with lymphomatous meningitis and sepsis.

Despite treatment with Meropenem, Vancomycin, Dexamethasone, Levetiracetam, G‐CSF, and vitamin B‐complex, the patient's condition deteriorated. She developed bilateral fixed mydriasis, indicating brain death. With severe pancytopenia (WBC = 0.3 × 10^9^/L [normal = 4.5–11.0 × 10^9^/L], Hb = 9.9 g/dL [normal = 9.5–15.5 g/dL], platelets = 7000/μL [normal = 150 000–400 000 /μL]) and despite intensive care, the patient passed away in the ICU on February 28, 2023.

**FIGURE 3:**
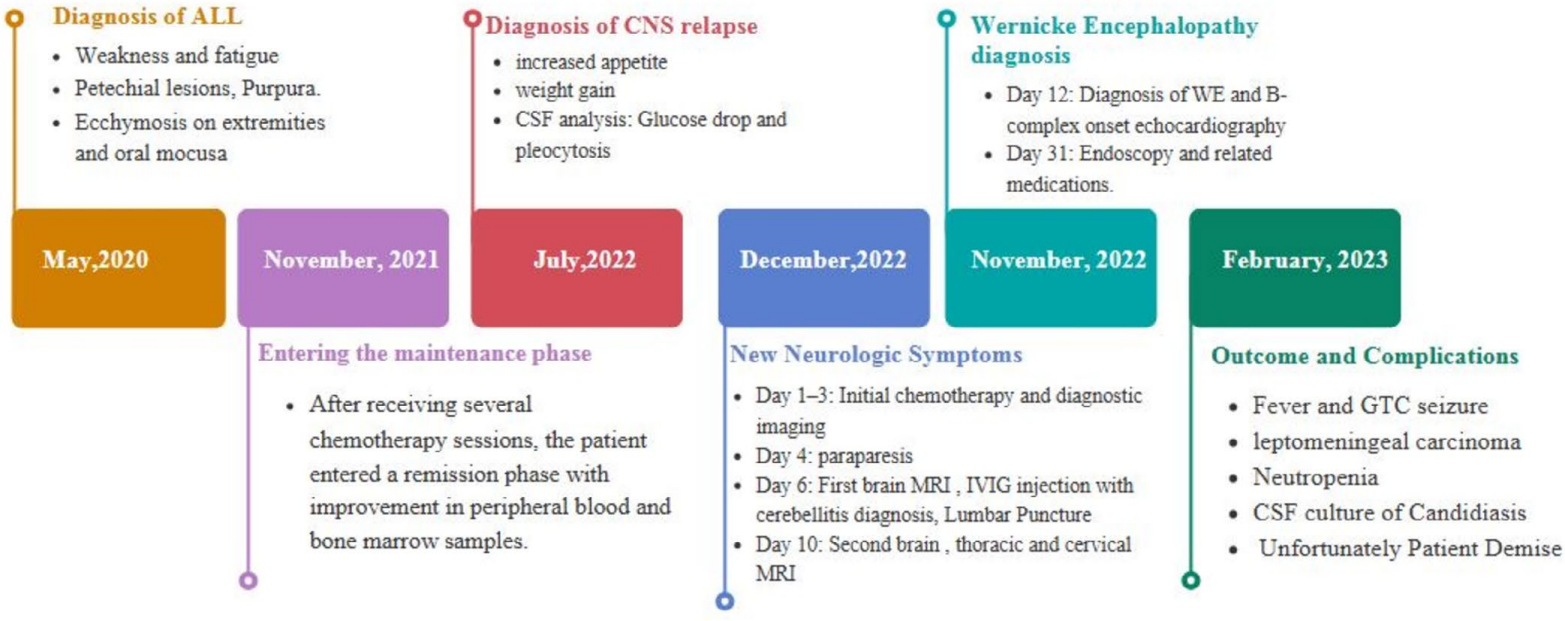
Timeline of the patient's hospitalization and key clinical events.

The timeline of the patient's symptoms, medical investigations, and treatments is presented in Figure 3.

## 3 Discussion

Thiamine deficiency depletes thiamine diphosphate (TDP), disrupting the pentose‐phosphate pathway and TCA cycle, and leading to the neuropsychiatric syndrome known as WE. This leads to lower ATP, NADPH, DNA, and RNA, which makes the cell more sensitive to oxidative stress. Cytotoxic edema results from decreased cellular pH and electrolyte imbalances due to toxic substances like glutamate and lactate. Vasogenic edema occurs from astrocyte damage related to reduced ATP, pH changes, and oxidative stress, affecting the blood–brain barrier (BBB) disorder [22, 23, 24, 25].

Thiamine deficiency symptoms vary, affecting the cardiovascular and neurological systems. It can lead to heart failure, and severe cases may cause WE. Long‐term mild to moderate deficiency leads to peripheral polyneuropathy [26]. Subclinical thiamine deficiency in children presents nonspecific symptoms like irritability, abdominal discomfort, headaches, and failure to thrive [27].

Other causes include excessive alcohol use, bariatric surgery, malignancies (especially GI cancers), and so on [3]. Many patients have malignancies, often due to nutrient deficiencies from nausea, vomiting, total parenteral nutrition (TPN), and so on [28]. Nausea and vomiting after CNS relapse can cause weight loss and contribute to other complications. Leukemia drugs like MTX affect folate metabolism, while asparaginase disrupts protein synthesis, exacerbating these issues. Diagnosing WE in children with ALL is challenging due to its rarity compared to adults. Neurological symptoms may be caused by CNS involvement or treatment.

### 3.1 Imaging Findings and Interpretation

CT scan findings are usually negative in the acute phase but may show reduced attenuation density in medial thalami and periaqueductal gray matter. Due to MRI's high sensitivity, reaching up to 93%, this imaging modality is the preferred diagnostic tool, and it usually shows bilateral lesions mainly in the hypothalamus, paraventricular thalamus, periaqueductal region, mammillary bodies, and 4th ventricle. Uncommon lesions can be seen in the cerebellum [29], putamen, caudate, and cranial nerve nuclei [2]. In pediatric WE, MRI frequently reveals symmetrical signal abnormalities, particularly within the thalami and mammillary bodies. These radiologic features have demonstrated a diagnostic sensitivity of approximately 85.2%, underscoring the utility of MRI in supporting clinical suspicion [30].

Our case demonstrated typical MRI features of WE, including T2/FLAIR hyperintensities around the third and lateral ventricles, periaqueductal gray, and atypically, the cerebellum. This atypical finding, though rare, has been previously described in pediatric WE. Studies have emphasized that atypical findings are more frequent in nonalcoholic patients, especially in children [31]. The MRI performed during suspected lymphomatous meningitis provided a valuable opportunity to compare with the previous MRI. The absence of prior lesions indicated radiological resolution of WE, thus affirming treatment efficacy.

### 3.2 Treatment Regimen and Justification

Due to the absence of standardized pediatric dosing, a high‐dose regimen (500 mg IV TDS) was employed based on adult recommendations and pediatric expert opinion [26]. This approach aimed to prevent under‐treatment, which may worsen outcomes. Magnesium was co‐administered due to its synergistic role in thiamine metabolism.

### 3.3 Delay in Diagnosis and Its Implications

Despite starting IV thiamine immediately upon WE being suspected, a delay did occur due to initial diagnostic uncertainty and adherence to imaging protocols. Previous work has shown that diagnostic delays beyond 14 days can cause irreversible brain damage [32], and in nonalcoholic patients, delays of ≥ 18 days were linked to severe sequelae compared to mild deficits when treated earlier [33].

### 3.4 WE and Immunosuppression

Although some studies suggest an association between acute WE and increased vulnerability to infections such as sepsis, especially during the untreated phase [34], there is limited evidence to support sustained immune suppression following thiamine repletion. Therefore, while WE may have contributed transiently to overall vulnerability, the progression to lymphomatous meningitis and sepsis in this patient is more likely attributable to her underlying malignancy and profound chemotherapy‐induced immunosuppression.

### 3.5 Uniqueness of Our Patient

This case stands out due to several unique features not commonly reported in the literature on WE in acute ALL, including early cerebellar involvement on MRI, thorough exclusion of alternative infectious and metabolic etiologies, radiologic documentation of lesion resolution, and a complex clinical course that progressed to lymphomatous meningitis and sepsis. These aspects underscore the diagnostic and management challenges of overlapping CNS complications in pediatric ALL.

### 3.6 Initial Differential Diagnosis and Diagnostic Process

Given the patient's initial neurological symptoms, GBS was considered due to areflexia and ascending weakness. However, findings from CSF analysis and electrodiagnostic evaluations reduced the likelihood of GBS. Other differentials, such as metabolic encephalopathies, CNS infections, and paraneoplastic syndromes, were systematically excluded via extensive CSF panels (including viral, bacterial, and fungal) and negative metabolic screens.

Although WE is primarily diagnosed clinically, especially when the classic triad (ophthalmoparesis, ataxia, and mental status changes) is present, MRI was performed early due to our institutional protocol for new‐onset neurological symptoms in pediatric oncology patients. The atypical early findings of this imaging led us to miss the whole picture. We acknowledge that clinical judgment should take precedence, and early empirical thiamine therapy should be initiated when WE is suspected [30]. The delay in diagnosis was partly due to anchoring bias and the absence of early clinical suspicion of thiamine deficiency despite elements of the classic triad.

## 4 Conclusion

Consider WE in any pediatric oncology patient presenting with ataxia, ophthalmoplegia, or altered mental status. The presence of any part of the classic triad warrants empirical high‐dose thiamine, regardless of imaging results. MRI remains useful for diagnosis and follow‐up, especially when atypical features are present. Early recognition and treatment may not change the course of the underlying malignancy, but they significantly affect the neurological outcome and quality of life.

### 4.1 Patient's Perspective

Despite her illness, she showed remarkable resilience. Her daily life was significantly impacted, limiting her ability to participate in kindergarten. The treatment journey was challenging, including chemotherapy, different diagnostic tests, and side effects of disease and treatment. During her hospitalizations, she received compassionate care, allowing her to remain comfortable and near loved ones. While her parents were deeply shocked by her passing, the prolonged course of her illness and increased hospitalizations in recent months led them to anticipate her death to some extent. Her parents cherish the memories of those last moments and emphasize the importance of support and open communication with healthcare providers.

## Author Contributions

G.S. led the project, conducted the literature review, and wrote the main manuscript. M.D. contributed to patient data analysis, drafted sections of the case presentation, and reviewed the final manuscript. F.M. reviewed the clinical aspects, provided critical revisions, and ensured accuracy in data interpretation. S.S. provided feedback on therapeutic approaches and contributed to the discussion section. B.B. assisted with imaging analysis and provided minor feedback on the radiological interpretation. S.A. supervised the overall project, contributed to the conceptual design, and critically revised the manuscript for key intellectual content and is the corresponding author for any answers to the editors' questions. All authors read and approved the final manuscript.

## Consent

Written informed consent for the publication of this case report and accompanying images was obtained from the patient's parents after her death. A copy of the written consent is available for review by the Editor‐in‐Chief of this journal upon request.

## Conflicts of Interest

The authors declare no conflicts of interest.

## Data Availability

All data supporting the conclusions of this case report are included within the article.
